# TargetRNA2: identifying targets of small regulatory RNAs in bacteria

**DOI:** 10.1093/nar/gku317

**Published:** 2014-04-21

**Authors:** Mary Beth Kery, Monica Feldman, Jonathan Livny, Brian Tjaden

**Affiliations:** 1 Computer Science Department, Wellesley College, Wellesley, MA 02481, USA; 2 Broad Institute of MIT and Harvard, Cambridge, MA 02142, USA

## Abstract

Many small, noncoding RNAs (sRNAs) in bacteria act as posttranscriptional regulators of messenger RNAs. TargetRNA2 is a web server that identifies mRNA targets of sRNA regulatory action in bacteria. As input, TargetRNA2 takes the sequence of an sRNA and the name of a sequenced bacterial replicon. When searching for targets of RNA regulation, TargetRNA2 uses a variety of features, including conservation of the sRNA in other bacteria, the secondary structure of the sRNA, the secondary structure of each candidate mRNA target and the hybridization energy between the sRNA and each candidate mRNA target. TargetRNA2 outputs a ranked list of likely regulatory targets for the input sRNA. When evaluated on a comprehensive set of sRNA-target interactions, TargetRNA2 was found to be both accurate and efficient in identifying targets of sRNA regulatory action. Furthermore, TargetRNA2 has the ability to integrate RNA-seq data, if available. If an sRNA is differentially expressed in two or more RNA-seq experiments, TargetRNA2 considers co-differential gene expression when searching for regulatory targets, significantly improving the accuracy of target identifications. The TargetRNA2 web server is freely available for use at http://cs.wellesley.edu/∼btjaden/TargetRNA2.

## INTRODUCTION

In recent years, small noncoding RNA (sRNA) genes have been found to pervade bacterial genomes (1). The largest family of sRNAs corresponds to sRNAs that act as posttranscriptional regulators by base pairing with their message targets. Many of these base pairing sRNAs are *cis*-acting RNAs in that they are transcribed opposite to their target RNA (2). Because these sRNAs are antisense, at least in part, to their target, they share an extended region of complementarity to their target. In contrast to *cis*-acting sRNAs, the *trans*-acting sRNAs typically have limited complementarity to their mRNA regulatory targets. As in the case of microRNAs in eukaryotes, *trans*-acting sRNA regulators in bacteria that bind via base pairing to messages typically affect the translation and stability of their targets. Commonly, these sRNAs inhibit the translation of their mRNA target, e.g. by binding in the neighborhood of the translation initiation site and blocking ribosome binding. Additionally, these sRNAs may decrease the stability of the message and target it for degradation by RNase E (3,4). Less commonly, sRNAs can activate translation, e.g. by freeing translation initiation sites that would otherwise be occluded by an inhibitory secondary structure (5,6). The products of an sRNA gene may interact with multiple mRNAs (7), enabling sRNAs to effect global regulatory responses. While the number of identified sRNAs in bacteria has exploded in recent years, thanks in part to advances in high-throughput sequencing technology, the bottleneck has quickly become, not identifying these sRNAs, but elucidating their regulatory targets (8). A number of experimental approaches have been employed for the purpose of large-scale target identification (9,10), yet these methods do not scale with the rapid increase in identified sRNAs. Thus, computational methods, which can be more efficient than experimental approaches, may be used as a first step in helping characterize targets of *trans*-acting regulatory RNAs in bacteria. TargetRNA2 is one such computational approach—a web server freely available for use, designed to identify mRNA targets, accurately and efficiently, of *trans*-acting sRNAs in bacteria.

TargetRNA2 builds on several other web servers that exist for biocomputational prediction of sRNA regulatory targets in bacteria (reviewed in (11)). The first web server designed specifically for bacterial sRNA target identification, TargetRNA (12), predicts sRNA targets using a straightforward hybridization model for sRNA-target interactions as well as a seed region comprised of a short series of consecutive base pairs between the two RNAs. TargetRNA also offers predictions of orthologous sRNA-target interactions in related bacteria, though the orthologous interactions are not used as a feature in target prediction. IntaRNA (13) predicts sRNA targets by combining the hybridization energy from intermolecular base pairings of sRNA-target interactions with the energy associated with the interacting regions being unpaired in intramolecular structures. IntaRNA also incorporates seed regions composed of consecutive base pairs into its target interaction predictions. RNApredator (14), built upon RNAplex (15), considers intermolecular interaction energies when predicting sRNA targets and uses an affine function for structural loop sizes rather than a more common logarithmic function in order to increase its efficiency. Peer and Margalit (16) approach the problem of predicting sRNA-target interactions from a different perspective; rather than focusing on targets, they investigate the target binding regions of sRNAs and find that sRNA interaction sites are better conserved and more accessible structurally than other regions of the sRNA, suggesting that biocomputational tools should emphasize conserved and accessible sRNA regions when predicting sRNA-target interactions.

TargetRNA2 combines many of the features from the above-mentioned tools into a fast and accurate target identification system. Specifically, TargetRNA2 uses four primary features for sRNA target identification. The first feature is conservation of the sRNA. The input sRNA sequence is compared to every sequenced replicon available in RefSeq (17). Regions of the sRNA sequence that show greater conservation are considered more likely to be target interacting regions. The second feature is accessibility of the sRNA. The ensemble of sRNA structures is considered. Each sRNA structure in the ensemble is weighted by its stability. Those regions of the sRNA that are more accessible throughout the weighted ensemble are considered more likely to be target interacting regions. The third feature is accessibility of the mRNA. For each candidate mRNA target, the ensemble of its structures is considered. Each mRNA structure in the ensemble is weighted by its stability. Those regions of the mRNA that are more accessible throughout the weighted ensemble are considered more likely to be sRNA interacting regions. The fourth feature is energy of hybridization. mRNAs that have one or more regions with low hybridization energy to one or more regions of the sRNA are considered more likely to be targets of the sRNA. The TargetRNA2 web server provides a user-friendly interface for target identification based on the abov-ementioned four features.

Since sRNA-target interactions are best understood in *Escherichia coli*, TargetRNA2's performance was evaluated on a comprehensive set of 105 experimentally verified interactions in *E. coli* (16). TargetRNA2 was found to outperform other approaches in its ability to identify sRNA targets accurately. TargetRNA2 also has the advantage of being more efficient, requiring only seconds to execute, in contrast to other approaches that require minutes or hours. Furthermore, TargetRNA2 has the unique ability among sRNA target prediction systems to integrate RNA-seq data in order to improve prediction results. As an additional feature in target prediction, TargetRNA2 optionally considers genes that are co-differentially expressed with an sRNA in two or more RNA-seq experiments. Using data from a variety of RNA-seq experiments in *E. coli*, where various sRNAs are differentially expressed, we find that TargetRNA2's target prediction performance improves dramatically when co-differential expression is considered.

## TARGETRNA2 WEB SERVER

The TargetRNA2 web server takes, as input, the nucleotide sequence corresponding to an sRNA and the name of an annotated bacterial replicon. TargetRNA2 considers each mRNA in the replicon as a possible target of the sRNA. For each candidate mRNA target, TargetRNA2 focuses its search for an sRNA–mRNA interaction in a neighborhood around the ribosome binding site of the mRNA, since most documented interactions occur within the 5′ untranslated region of the mRNA or near the beginning of the mRNA coding sequence. TargetRNA2 will not consider interactions outside of this neighborhood, so the size and location of the neighborhood are user-adjustable parameters.

TargetRNA2 uses four features to identify likely sRNA regulatory targets. The first feature is conservation of regions of the sRNA sequence. BLASTN (18) is used to compare the sRNA sequence to every bacterial replicon available in RefSeq (17). For all sequences found to be significantly similar to that of the sRNA, a multiple sequence alignment is performed using ClustalW2 (19). From the multiple sequence alignment, positional entropies are computed that are used to identify regions of the sRNA sequence that are highly conserved (16). More highly conserved regions are considered by TargetRNA2 more likely to be target interacting regions (16). The second feature is accessibility of regions in the sRNA secondary structure. RNAfold from the Vienna RNA Package (20) is used to determine the probability that regions of the sRNA sequence are accessible, i.e. unpaired, in the ensemble of secondary structures. More accessible regions are considered by TargetRNA2 more likely to be target interacting regions (16). The third feature is accessibility of regions in the mRNA secondary structure. RNAplfold (21) is used to determine the probability that regions of the mRNA sequence are accessible, i.e. unpaired, in the ensemble of secondary structures. More accessible regions are considered by TargetRNA2 more likely to be target sites (13). The fourth feature is the energy of hybridization between the sRNA and candidate mRNA target. RNAduplex (20) is used to determine regions of the sRNA that have low hybridization energy with regions of a candidate mRNA target. Optionally, TargetRNA2 can restrict its target search to a user-specified subset of genes. This option was used, for example, to search for targets of sRNAs in *E. coli* that are co-differentially expressed with the sRNA in RNA-seq experiments (see Results).

**Figure 1.**
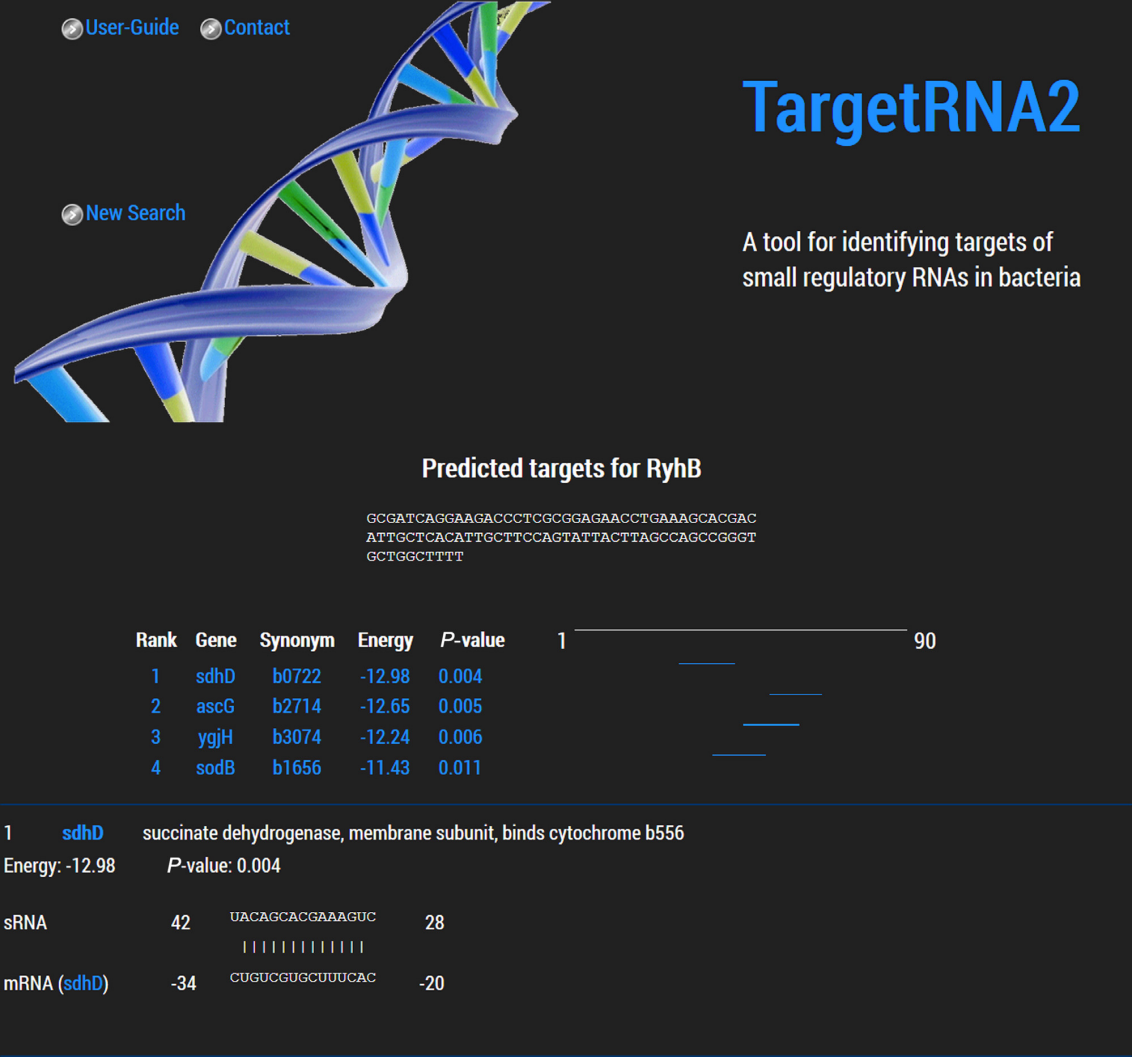
Sample output from TargetRNA2's search for regulatory targets of the sRNA RyhB in *E. coli* is illustrated. For brevity, only 4 of 21 significant (*P*-value < 0.05) targets identified by TargetRNA2 are shown in the figure. Below the RyhB sRNA sequence, TargetRNA2's output begins with a ranked list of candidate targets, including thermodynamic energy (kcal/mol) of hybridization between the two RNA molecules as well as a *P*-value indicating the probability of an interaction occurring by chance that is at least as energetically favorable. The short blue line to the right of each list item is a graphical representation of where the interaction occurs within the sRNA. Below the list, detailed information about each identified interaction is shown (in the figure, detailed information is shown only for *sdhD*, the first of the identified targets), including a graphic depiction of the interaction, the precise coordinates of the interaction and a link to a web page at the National Center for Biotechnology Information (NCBI) with more specifics about the gene target.

After searching all mRNAs in the specified replicon for interactions with the sRNA, TargetRNA2 outputs a list of likely regulatory targets ranked by *P*-value. The *P*-value for a target corresponds to the likelihood of observing as strong an interaction by chance. For each target identified by TargetRNA2, a graphical depiction of the sRNA–mRNA interaction is shown along with information about the message's product and a link to the corresponding gene page from RefSeq (17), as shown in Figure 1.

## RESULTS

Since sRNA and target interactions are best understood in *E. coli*, TargetRNA2's performance was evaluated on a comprehensive set of 105 experimentally verified interactions from 24 sRNAs in *E. coli* (16). TargetRNA2's performance was compared to that of other leading approaches: IntaRNA (13), RNApredator (14) and TargetRNA (22). Table 1 lists verified interactions that are identified by the different approaches at the same false positive rate of 0.9%, i.e. when the approaches each generate the same number of predictions. As shown in Table 1, TargetRNA2 identifies more verified interactions than the other approaches.

**Table 1.** For 105 target interactions involving 24 sRNAs in *E. coli*, the tables indicates whether the interaction was identified by each of four different computational approaches when the false positive rate is limited to 0.9%. TargetRNA2 identifies 28 interactions, IntaRNA identifies 26 interactions, TargetRNA identifies 20 interactions and RNApredator identifies 14 interactions.

sRNA	mRNA Target	Identified by TargetRNA2	Identified by IntaRNA	Identified by TargetRNA	Identified by RNApredator
ArcZ	rpoS				
ArcZ	sdaC				
ArcZ	tpx				
ChiX	chiP	Yes	Yes		Yes
ChiX	dpiB	Yes			Yes
CyaR	luxS				
CyaR	nadE				
CyaR	ompX				
CyaR	yqaE				
DicF	ftsZ				
DsrA	hns	Yes	Yes	Yes	Yes
DsrA	rpoS				Yes
FnrS	cydD	Yes			
FnrS	folE				
FnrS	folX				
FnrS	gpmA				
FnrS	maeA				
FnrS	metE				
FnrS	sodA				
FnrS	sodB				
FnrS	yobA		Yes	Yes	
GcvB	argP				
GcvB	argT	Yes	Yes		
GcvB	brnQ	Yes			
GcvB	cycA	Yes	Yes	Yes	
GcvB	dppA	Yes	Yes	Yes	
GcvB	gdhA	Yes	Yes		
GcvB	ilvC	Yes	Yes	Yes	Yes
GcvB	ilvE	Yes	Yes		
GcvB	livJ	Yes	Yes		
GcvB	livK	Yes	Yes	Yes	Yes
GcvB	lrp	Yes	Yes		
GcvB	metQ	Yes	Yes		
GcvB	oppA	Yes	Yes	Yes	
GcvB	serA	Yes	Yes	Yes	
GcvB	sstT				
GcvB	thrL	Yes			
GcvB	ybdH	Yes	Yes	Yes	
GlmZ	glmS				
IstR	tisB				Yes
MgrR	eptB				
MgrR	ygdQ		Yes	Yes	
MicA	fimB			Yes	
MicA	gloA				
MicA	lamB		Yes		
MicA	ompA		Yes	Yes	
MicA	ompW				
MicA	ompX				
MicA	phoP			Yes	
MicA	tsx				
MicA	yfeK				
MicC	ompC	Yes	Yes	Yes	Yes
MicF	ompF		Yes	Yes	Yes
OhsC	shoB				Yes
OmrA	cirA				
OmrA	csgD				
OmrA	fecA				
OmrA	fepA				
OmrA	ompR				
OmrA	ompT		Yes		
OmrB	cirA				
OmrB	csgD				Yes
OmrB	fecA				
OmrB	ompR				
OmrB	ompT				
OxyS	fhlA				
OxyS	rpoS				
RprA	rpoS				
RseX	ompA				
RseX	ompC	Yes			
RybB	fadL				
RybB	fimA				
RybB	fiu				
RybB	hinT				
RybB	lamB			Yes	
RybB	ompA				
RybB	ompC				
RybB	ompF				
RybB	ompW				
RybB	rbsB				
RybB	rluD				
RybB	tsx				
RybB	ycfL				
RybB	ydeN				
RybB	yfeK				
RydC	yejA				
RyhB	acnA	Yes			
RyhB	bfr				
RyhB	cysE	Yes	Yes	Yes	
RyhB	ftnA				
RyhB	fumA		Yes		
RyhB	fur				
RyhB	iscS				
RyhB	sdhD	Yes		Yes	
RyhB	shiA	Yes			Yes
RyhB	sodB	Yes		Yes	
SgrS	manX				
SgrS	ptsG	Yes	Yes		Yes
Spot42	fucI				
Spot42	galK	Yes	Yes	Yes	
Spot42	gltA				
Spot42	nanC				Yes
Spot42	srlA				
Spot42	sthA				
Spot42	xylF				

**Figure 2.**
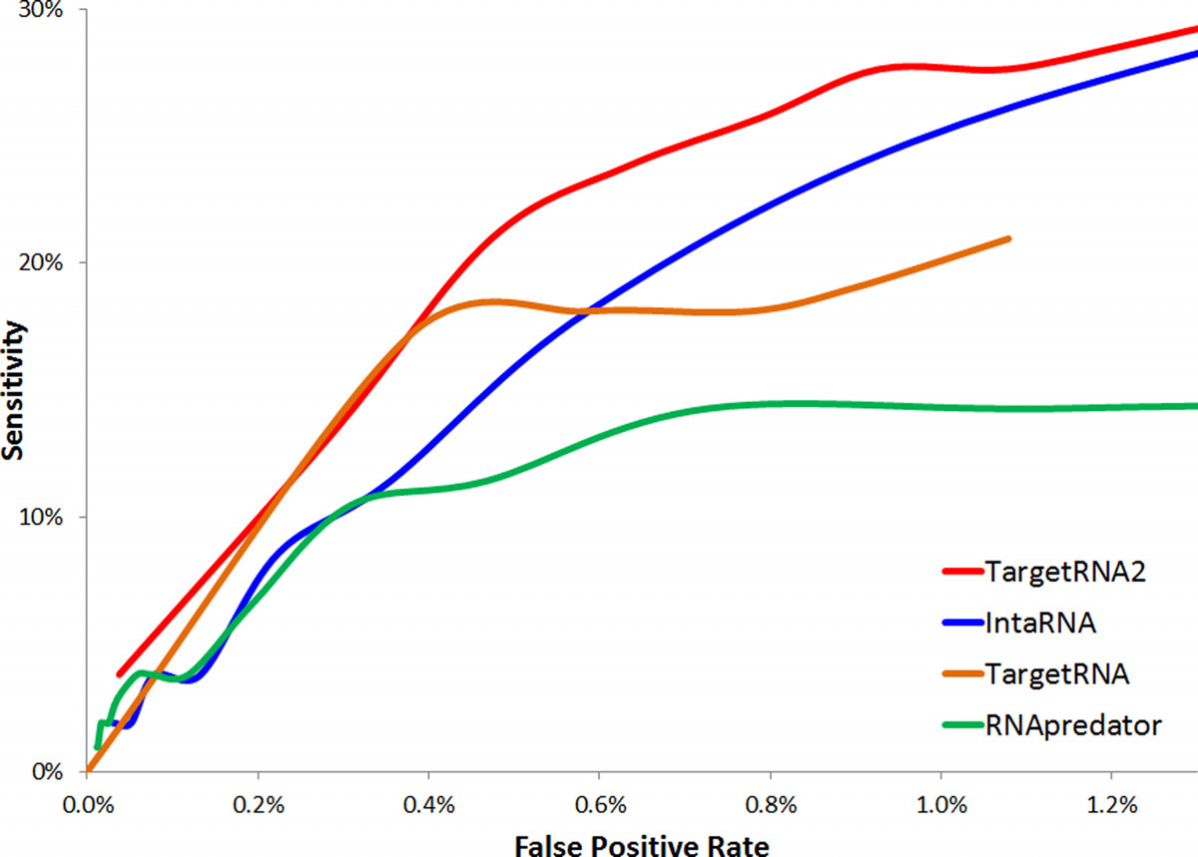
Receiver operating characteristic curves indicating the performance of TargetRNA2 as well as three other target identification systems: IntaRNA (13), TargetRNA (12) and RNApredator (14). The curves are based on 105 verified interactions of 24 sRNAs in *E. coli* (16). The *x*-axis represents the percentage of noninteractions that each system predicted as likely interactions. The *y*-axis represents the percentage of verified interactions correctly identified by each system. Each point along a curve represents the sensitivity and false positive rate of a system's identifications at a specified *P*-value (TargetRNA2, IntaRNA and TargetRNA) or *z*-score (RNApredator).

In order to evaluate the parameter space more broadly and explore the trade-off between sensitivity and false-positive rate, we then proceeded to assess the performance of the approaches using different *P*-value or *z*-score thresholds. It should be noted that any target prediction that does not correspond to an experimentally verified target is considered a ‘false positive’ here, though many of these predictions may well correspond to as yet unverified targets. Thus, the reported false positive rate should be considered an upper bound. As shown in Figure 2, TargetRNA2 outperforms the other methods, consistently identifying more true interactions (higher sensitivity) while making fewer spurious predictions (lower false-positive rate).

The efficiency of TargetRNA2 was also considered. The mean execution time across the 24 sRNAs in *E. coli* (16) was calculated for TargetRNA2 and for two alternative approaches. As shown in Table 2, TargetRNA2 is dramatically faster than other approaches, generally requiring only seconds, as opposed to minutes, to execute. TargetRNA2's speed has the benefit of enabling users to explore different parameter settings while performing repeated searches without long delays between each search.

**Table 2.** The table indicates the mean execution time, for 24 sRNAs in *E. coli*, of three different target identification systems.

System	Average execution time per sRNA
TargetRNA2	9 s
RNApredator	247 s
IntaRNA	4294 s

sRNA-mediated regulation often alters the steady-state abundance of target mRNAs. TargetRNA2, therefore, has the unique ability among sRNA target identification approaches to integrate RNA-seq data in order to improve its performance. As an additional feature when identifying targets, TargetRNA2 optionally considers genes that are co-differentially expressed (either positively or negatively) with an sRNA in two or more RNA-seq experiments. To evaluate the effect of incorporating co-expression patterns into sRNA target identifications, RNA-seq data derived from *E. coli* cultures grown in triplicate under various conditions (23) was used to identify targets for five sRNAs whose expression changed significantly between at least two of the conditions tested and for which interactions with target mRNAs are known: GcvB, MicA, RybB, SgrS and ArcZ. For each of these five sRNAs, only those mRNAs that were differentially expressed (*P*-value < 0.01) in the same pair of conditions as the sRNA were retained (23). The five sRNAs have 46 validated targets. Of these 46 targets, 29 are differentially expressed in the RNA-seq data in the same pair of conditions as their sRNA regulator. Ignoring the RNA-seq data, TargetRNA2 achieves a sensitivity of 35% for all 46 targets of these 5 sRNAs and a false positive rate of 0.77%. When RNA-seq is incorporated, TargetRNA2 achieves a sensitivity of 48% for the 29 differentially expressed targets and 30% for all 46 targets of these 5 sRNAs and a false positive rate of 0.37%. When RNA-seq is incorporated into TargetRNA2, the dramatic reduction in the false positive rate, by more than half, is notable since limiting the number of false positive predictions has been a major challenge for target identification systems. Interestingly, TargetRNA2's performance was especially strong for two sRNAs (GcvB and SgrS) identifying 16 out of 19 verified targets and especially poor for three sRNAs (MicA, RybB, ArcZ) identifying 0 out of 27 targets. TargetRNA2's poor performance in identifying targets for MicA, RybB and ArcZ is consistent with that of other approaches IntaRNA, TargetRNA and RNApredator, which identified 2 out of 27 targets, 4 out of 27 targets and 0 out of 27 targets, respectively (Table 1).

## DISCUSSION

With the rapidly expanding number of bacterial sRNA regulators that are being identified, thanks in part to advances in RNA-seq technology, comes the need for approaches that can characterize targets of these sRNA regulators. Computational methods for identifying regulatory targets of sRNA action offer a good first step, in that they are scalable and they can efficiently provide a small set of candidate targets, which can then be followed up on through more focused experimental methods. TargetRNA2 is a web server that enables rapid and accurate identification of targets of bacterial sRNA action.

TargetRNA2 uses several features to identify message targets of sRNA regulation, including conservation of regions of the sRNA, structural accessibility of regions of the sRNA, structural accessibility of regions of the mRNA and energy of hybridization between the two RNAs. When compared to other computational approaches, TargetRNA2 offers improved performance both in terms of the accuracy of its predictions and the speed of its execution. TargetRNA2 is also unique among computational target identification methods in that it allows for incorporation of RNA-seq data. Our results using TargetRNA2 with RNA-seq data suggest that there is significant value in integrating RNA-seq data into target identification systems, particularly in reducing false positive rates, which has been a major challenge for computational identification of sRNA targets. This feature of TargetRNA2 will become increasingly useful as the number of RNA-seq data sets from diverse bacteria continues to grow. We offer the TargetRNA2 web server in the hope that it will be a useful resource for those interested in RNA regulators in bacteria.
